# Antioxidant and Photoprotective Capacity of Secondary Metabolites Isolated from *Pseudocyphellaria berberina*

**DOI:** 10.3390/molecules30183833

**Published:** 2025-09-22

**Authors:** Cecilia Rubio, Javiera Ramírez, José L. Rojas, Norma A. Valencia-Islas, Carolina Campos, Natalia Quiñones

**Affiliations:** 1 Herbario de Líquenes, Escuela de Química y Farmacia, Facultad de Farmacia, Universidad de Valparaíso, Valparaíso 2340000, Chile; cecilia.rubio@uv.cl (C.R.); javiera.ramirez@uv.cl (J.R.); 2 Magíster en Gestión Farmacéutica y Farmacia Asistencial, Escuela de Química y Farmacia, Facultad de Farmacia, Universidad de Valparaíso, Valparaíso 2340000, Chile; 3 Departamento de Química, Facultad de Ciencias, Universidad Nacional de Colombia, Sede Bogotá 111321, Colombia; jlrojasa@unal.edu.co; 4 Departamento de Farmacia, Facultad de Ciencias, Universidad Nacional de Colombia, Sede Bogotá 111321, Colombia; navalenciai@unal.edu.co; 5 Laboratorio de toxicología (LADETOX), Escuela de Química y Farmacia, Facultad de Farmacia, Universidad de Valparaíso, Valparaíso 2340000, Chile; carolina.campos@uv.cl; 6 Centro de Investigación, Desarrollo e Innovación de Productos Bioactivos (CInBIO), Universidad de Valparaíso, Valparaíso 2340000, Chile

**Keywords:** *Pseudocyphellaria berberina*, antioxidant activity, photoprotection

## Abstract

Exposure to sunlight, whose main component is UV radiation (UVR), leads to various skin damage such as sunburns, premature aging, or more severe issues such as increased symptoms of autoimmune disease and skin cancer. Therefore, there is a growing interest in developing improved photoprotective agents that can protect skin from sunlight incidence and antioxidants that counteract the oxidative stress caused by it. Lichens are a source of such agents since they adapt to extreme environments including those with high UVR by biosynthesizing metabolites with those properties. In this study, brialmontin 2 (**1**), physciosporin (**2**), and pseudocyphellarin A (**3**) were isolated for the first time from the lichen *Pseudocyphellaria berberina* (G. Forst.) D. J. Galloway & P. James, along with calycin (**4**) and 22-hydroxystictan-3-one (**5**). Their structural characterization was carried out by spectroscopy (^1^H and ^13^C NMR). Sun protection factor (SPF) along with critical wavelength (λ_crit_), a UVA/UVB ratio (UVA/UVB-r) of **one** to **five,** and acetone extract (**AE**) were evaluated spectrophotometrically as a measure of their UVB and UVA photoprotective capacities, respectively. Additionally, their antioxidant activity was measured by scavenging DPPH free radicals (RSA). Compounds **2**, **4,** and **AE** showed “medium” UVB photoprotective capacities (with SPFs between 15 and 30). Additionally, **4** and **AE** presented “maximum” UVA photoprotective capacities (λ_crit_ > 370 nm and UVA/UVB-r > 0.8), whereas this activity was “good” for **2** and **3** (λ_crit_ 350 to 370 nm and UVA/UVB-r 0.4 to 0.6), and “moderate” for **1** (λ_crit_ 335 to 350 nm and UVA/UVB-r 0.2 to 0.4). All compounds and **AE** showed antioxidant activity, standing out were **AE** and **4** with activity comparable to the controls (ca. 95 and 81 RSA %, respectively, at 1000 ppm). **AE** and **4** are dual agents with photoprotective (UVB-UVA) and antioxidant capacities that could help prevent skin damage associated with sunlight. In silico assays suggest that **4** spontaneously diffuses into the stratum corneum with limited absorption through the skin. Additionally, **4** lacks potential toxicity to Normal Human Epidermal Keratinocytes (showing viability ca. 70% at 100 ppm); therefore, it is a candidate for the development of sunscreen formulations.

## 1. Introduction

Skin cancer is a public health concern, it is already one of the most common cancers worldwide, and its incidence is increasing [1,2]. In 2020, there was an estimated 1.5 million new cases. Its etiology is due to factors such as chronic sun exposure, artificial exposure to UV radiation (UVR), type of skin and eye tone, family history of skin cancer, and certain diseases [3,4].

Chronic sun exposure can damage skin, and this can lead to cancer. It is currently known that both UVA and UVB radiation can cause skin cancer. Exposure to UVB radiation generates the formation of pyrimidine-pyrimidone photoproducts and pyrimidine dimers capable of damaging DNA [5,6]. Additionally, chronic exposure to UVA radiation can generate reactive oxygen species (ROS) that also contribute to the development of this cancer [7]. In this context, antioxidants play a crucial role in human medicine. These compounds act by neutralizing reactive oxygen species (ROS), which are generated both by exogenous sources such as UV radiation and by endogenous cellular processes. The accumulation of ROS has been linked to chronic diseases such as cancer, cardiovascular diseases, neurodegenerative diseases, and inflammatory processes, making the search for effective antioxidants a therapeutic and preventive priority in multiple areas of biomedicine [7,8].

To reduce the risk of developing skin cancer, sunscreen formulations containing photoprotective compounds have been developed to reduce and prevent the damage associated with UVR [9,10,11]. However, the currently available sunscreens present problems such as poor water resistance and photoinstability by the formation of harmful byproducts. Also, they may have a low molar absorption of UVA and UVB radiation [12]. Therefore, it is relevant to search for new molecules that can overcome these limitations and simultaneously present antioxidant capacity to counteract ROS.

Aromatic organic compounds, both of natural and synthetic origin, appear as good alternatives to overcome this problem because they show photoprotective and antioxidant properties as result of absorbing photons emitted by UVR and stabilizing free radicals [10,13]. In the search for these compounds, lichens are an original source of bioactive molecules with these characteristics. Lichens are symbiotic organisms formed by a photobiont (algae or/and cyanobacteria) and a mycobiont. They biosynthesize secondary metabolites that are classified according to their biosynthetic pathways as follows: monocyclic phenols; depsides; depsidones; depsones; debenzofurans; xanthones; naphtoquinones and anthraquinones (polyketide pathway); steroids and carotenoids (mevalonic acid pathway); as well as amino acid derivatives and cyclopeptides (shikimic acid pathway) [14,15]. Many of these metabolites have great potential as bioactive molecules showing a broad range of biological activities, among which anticancer and antimicrobial activities stand out [8,16,17,18].

In addition, lichens adapt to stressful conditions, such as increased UVR [18], biosynthesizing photoprotective pigments such as usnic acids [19], depsidones, dibenzofurans, and anthraquinones, which protect against UVB radiation, or xanthones and pulvinic acid derivatives, which protect against UVA radiation [19,20].

*Pseudocyphellaria berberina* (G. Forst.) D. J. Galloway & P. James is an endemic species of South America, epiphytic in *Nothofagus* spp. It is found in open habitats with high humidity, rain, and high luminosity; therefore, it is expected that it biosynthesizes photoprotective and/or antioxidant metabolites. *P. berberina* has a yellow medulla and a green photobiont. It is characterized as rosette-forming to irregularly spreading in extensive wards, and is grown on the bark of trees and sometimes on rocks. In Chile, it is distributed in Juan Fernández Archipelago, and from Mocha Island (VIII Region) to Tierra del Fuego (XII Region) [21].

In this study, the lichen *P. berberina* was selected as a source of natural compounds that could be used in the dermo-cosmetic field as ingredients of sunscreen formulations. The rationale for selecting *P. berberina* lies in its ecological adaptation to UV stress, its endemic availability, and its known production of unique bioactive secondary metabolites that are not widely distributed outside the lichen kingdom.

Therefore, the isolation of its secondary metabolites along with the evaluation of their photoprotective and antioxidant activities were carried out. Additionally, some physicochemical parameters indicating the optimal applicability of the isolates on the skin were estimated in silico along with a determination of their potential cytotoxicity.

## 2. Results

### 2.1. Chemistry

**Figure 1:**
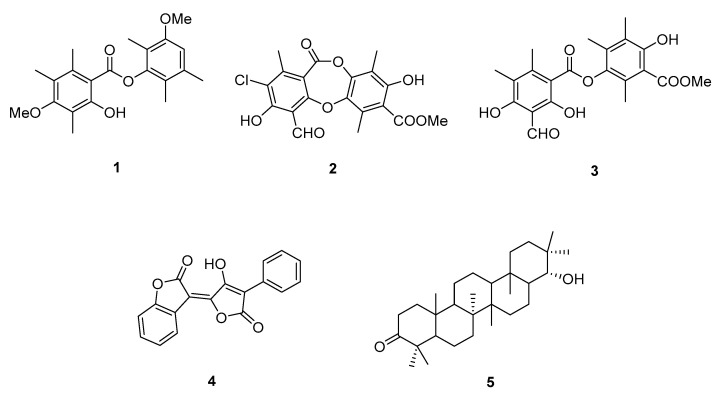
Isolated metabolites from *P. berberina.* Brialmontin 2 (**1**), physciosporin (**2**), pseudocyphellarin A (**3**), calycin (**4**), and 22-hydroxystictan-3-one (**5**).

Brialmontin 2 (**1**), physciosporin (**2**), pseudocyphellarin A (**3**), calycin (**4**), and 22- hydroxystictan-3-one (**5**) (Figure 1) were isolated from *P. berberina.* The compounds were purified by column chromatography and characterized by NMR spectroscopic techniques (^1^H-NMR and ^13^C-NMR) and physical properties. The spectroscopic data coincide with those reported in the literature [22,23]. Compounds **1** to **3** are reported for the first time in this study for *P. berberina* [24].

### 2.2. Photoprotective Activity

**Figure 2:**
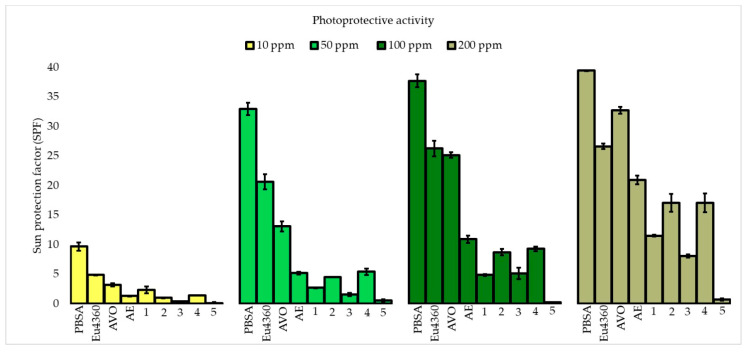
UVB photoprotective activity of extract of *P. berberina* and its isolated metabolites **1** to **5**. Data are presented as mean ± standard deviation (SD) from three independent experiments (n = 3).

The photoprotective activities of compounds **1** to **5** and **AE** were determined by spectrophotometric assays using 2-phenyl-5-bencimidazolesulphonic acid (PBSA), Eusolex 4360 (Eu4360), and avobenzone (AVO) as controls. Sun protection factor (SPF) was evaluated as a measure of UVB photoprotective activity. Furthermore, critical wavelength (λ_crit_) and UVA/UVB-r were evaluated as a measure of UVA photoprotective activity (Figure 2, Table 1).

Controls as well as lichen metabolites **1** to **4** and **AE** showed a concentration dependence on their SPF (*p* < 0.05), reaching their highest values at 200 ppm (Figure 2). PBSA evidenced its “high” UVB photoprotective capacity at 50 ppm (SPF 30 to 50) (*p* < 0.05). In turn, AVO and Eu4360 showed lower SPF values than PBSA along the studied concentration range according to their UVA and UVA-UVB properties, respectively.

The UVA filter AVO demonstrated **** or a “maximum” UVA photoprotective capacity (λ_crit_ ≥ 370 nm and UVA/UVB-r ≥ 0.8). In turn, Eu4360 exhibited *** or “good” UVA photoprotective activity (λ_crit_ 350 to 370 nm and UVA/UVB-r 0.4 to 0.6). In contrast, PBSA showed * or “no UVA” photoprotective activity (λ_crit_ 325 to 335 nm and UVA/UVB-r < 0.2) due to its UVB nature. **AE** and metabolite **4** were the most active UVA photoprotectors (Table 1).

### 2.3. Antioxidant Activity

**Figure 3:**
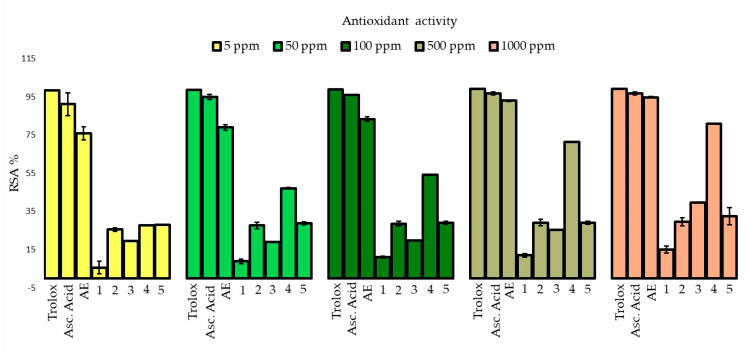
DPPH radical scavenging activity of the extract of *P. berberina* and its isolated metabolites **1** to **5**. Results are expressed as scavenging DPPH free radicals percentage (RSA%). Data are presented as mean ± standard deviation (SD) from three independent experiments (n = 3).

The antioxidant potential of compounds **1** to **5** and **AE** was evaluated through DPPH (2,2-diphenyl-1-picrylhydrazyl) radical scavenging activity. Trolox and ascorbic acid were used as controls (Figure 3).

**AE** and **4** showed outstanding DPPH radical scavenging activity, with all tested concentrations reaching their highest antioxidant activity at 1000 ppm (ca. 95 and 81%, respectively). This activity was comparable to that of the controls Trolox and ascorbic acid (ca. 99 and 97%, respectively). The other compounds were less active (lower than 40%) at the same concentration.

### 2.4. Cytotoxicity

**Figure 4:**
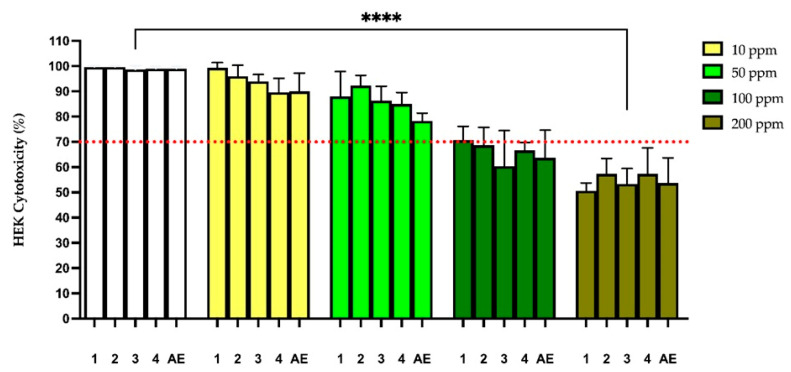
Cytotoxicity of compounds (**1** to **4**) and extract (**AE**) on HEK cells. Results are expressed as the percentage of viable cells relative to the untreated control group. Data are presented as mean ± standard deviation (SD) from three independent experiments (n = 3). Statistical significance was determined using one-way ANOVA followed by Tukey’s post-hoc test (**** *p* < 0.0001 compared to control).

To evaluate the preliminary safety of compounds **1** to **4** and **AE**, their cytotoxicity against Normal Human Epidermal Keratinocytes (HEKs) was determined using a resazurin metabolic viability assay. Figure 4 presents the results of the assay in a graph indicating the viability of keratinocytes exposed to different concentrations of the samples.

At a concentration of 100 ppm, which reports a good SPF (~10), an inhibition of viability of approximately 30% was observed for all the compounds and **AE**, which gives these molecules interesting potential as photoprotective agents. A concentration-dependent reduction in cell viability was observed for some compounds and the extract, particularly at 100 and 200 ppm.

### 2.5. In Silico Estimation of Skin Permeation and Application of **1** to **4** as Topical Dermatological Agents

To evaluate the possible skin permeation of **1** to **4** as well as their use as topical dermatological agents, physicochemical parameters, such as skin permeability coefficient (Log k_p_), topological polar surface area (TPSA), partition coefficient (cLogP), and Gibbs free energy of transfer (Δ_t_G°), as well as molecular weight (MW) and number of aromatic rings (Ar #) in their structure were estimated in silico (Table 2). Compound **5** was not evaluated due to its low photoprotective and antioxidant activities.

In addition, Gibbs free energy of transfer from aqueous to lipid phases (Δ_t_G°) and skin permeability coefficients (Log k_p_) preliminarily estimated that **1** to **4** and the photoprotective controls spontaneously diffuse into the stratum corneum, given their negative value as well as the fact that they would have limited skin penetration [27] (Table 2).

## 3. Discussion

In this study, the lichen *P. berberina* was selected as a source of natural photoprotective and antioxidant compounds that could be used in the dermo-cosmetic field as ingredients of sunscreen formulations. Brialmontin 2 (**1**), physciosporin (**2**), and pseudocyphellarin A (**3**) were isolated for the first time from *P. berberina* along with calycin (**4**), and 22-hydroxystictan-3-one (**5**) (Figure 1). The compounds were obtained in 0.27, 0.34, 1.62, 0.29, and 1.15% yields, respectively.

**AE** and compounds **1** to **5** were evaluated to determine their photoprotective (Figure 2) and antioxidant (Figure 3) capacities. **AE** showed a “low to medium” UVB photoprotective capacity from 50 to 200 ppm (with SPFs between 2 and 30), while this capacity was “low” for metabolites **1** and **3** (with SPFs between 2 and 15) (*p* < 0.05) and null for metabolite **5**, along the tested concentrations. In turn, metabolites **2** and **4** also showed “low to medium” UVB photoprotective capacities along the tested concentrations and they did not show significant differences in their values (*p* > 0.05). Considering the reference concentration for this test (200 ppm), **AE**, **2,** and **4** are photoprotectors with a “medium” UVB capacity (with SPFs between 15 and 30).

**AE** and metabolite **4** were the most active UVA photoprotectors (Table 1), showing **** and “maximum” scores (λ_crit_ ≥ 370 nm and UVA/UVB-r ≥ 0.8), followed by **3** and **2** with *** and “good” scores (λ_crit_ 350 to 370 nm and UVA/UVB-r 0.4 to 0.6) and **1** with ** and “moderate” activity (λ_crit_ 335 to 350 nm and UVA/UVB-r 0.2 to 0.4). In turn, **5** did not show UVA photoprotection.

**AE** and metabolites **2** and **4** are photoprotectors with UVB and UVA properties, whereas **1** and **3** are UVA photoprotectors. According to the Food and Drug Administration 2011 [28], **AE** and **4** are broad-spectrum photoprotectors because their λ_crit_ is ≥ 370 nm and their SPF is ≥15.

Regarding their antioxidant capacity, **AE** and metabolite **4** showed strong DPPH radical scavenging activity (RSA) (from 76 to 95 and 28 to 81 RSA%, respectively), along the evaluated concentrations. This added to their classification as broad-spectrum photoprotectors, make them candidates for advancing the development of dual dermatological agents. In addition, although metabolites **1** to **3** showed low antioxidant activity (lower than 40%), they also showed “low” to “medium” UVB and “moderate” to “good” UVA photoprotective activity; therefore, they could be considered as photoprotective ingredients in sunscreens formulations.

Compared to commercial UV filters, the compounds isolated in this study, particularly calycin (4), showed competitive performance in key parameters. Calycin is photostable [29], provides a medium SPF (15–30), and presented maximum UVA protection with a λcrit of 391.08 nm and a UVA/UVB ratio of 1.353, these values surpass those of commercial filters such as Eusolex 4360 and approach those of avobenzone (λcrit 378.44 nm, UVA/UVB = 1.74).

After a first glance of the toxic properties of **1** to **4** and **EA**, their cytotoxicity was estimated using Normal Human Epidermal Keratinocytes (HEKs). A concentration-dependent relationship was evident, especially for concentrations of 100 and 200 ppm. At a concentration of 100 ppm, where good photoprotective and antioxidant activity was observed, **1** to **4** were not significantly cytotoxic, showing viability ca. 70%.

Considering that **4** is a dual agent with broad spectrum photoprotective and antioxidant activities along with low cytotoxicity to HEKs, it can be considered a candidate for incorporation in combination with other sunscreens in dermo-cosmetic formulations for sun protection. Therefore, some physicochemical parameters indicating their feasibility at being formulated in topical dermatological dosage forms were estimated in silico along with those for compounds **1** to **3** (Table 2).

According to Santos et al., physicochemical parameters such as topological polar surface area (TPSA), molecular weight (MW), partition coefficient (cLogP), and number of aromatic rings (Ar#) are key for the initial selection of compounds aimed at being formulated in topical dermatological dosage forms [30]. Compounds accomplishing the following: TPSA ≤ 100 Å^2^, MW ≤ 500 Da, cLogP 1 to 4, and Ar# ≤ 2 will have an optimal performance as dermatological agents, being compatible with the most used excipients for these kinds of formulations. However, when two or more deviations are presented for such parameters, a more detailed evaluation should be made to not discard potential topical dermatological agents [30].

Metabolites **1** (TPSA, MW and Ar#), **3** (cLog P, MW and Ar#), and **4** (cLog P, TPSA and MW) complied with three of these parameters, whereas **2** only complied with two (cLog P and MW); therefore, **1**, **3,** and **4** could be added as photoprotective ingredients of sunscreens formulations, with these being compatible with the most used excipients for these kinds of formulations.

In addition, other physicochemical parameters such as Gibbs free energy of transfer from aqueous to lipid phases (Δ_t_G°) and skin permeability coefficients (Log k_p_) were also calculated for **1** to **4** to preliminarily estimate if these substances would accumulate in the stratum corneum (the lipophilic outermost layer of the epidermis) or if they would absorb through the skin (Table 2). A negative Δ_t_G° value suggests the spontaneous diffusion of a substance into the stratum corneum, whereas a negative Log k_p_ value suggests limited skin penetration, with a greater negative value indicating a reduced permeation potential [27].

Metabolites **1** to **4** and the photoprotective controls showed negative Δ_t_G° values, indicating that the diffusion of these substances into the stratum corneum occurs spontaneously. Additionally, their Log k_p_ values were also negative, indicating that they would have limited skin penetration [27]. Compound **4** had the most negative Log k_p_ value followed by **2**, **3,** and **1,** indicating that they would not be absorbed through the skin; therefore, they would have reduced systemic toxicity associated with their absorption.

Considering the photoprotective and antioxidant properties of metabolites **1** to **4**, along with their physicochemical parameters indicating skin permeation and low cytotoxicity, calycin (**4**) could constitute a UV filter for sunscreen formulations.

The percentage obtained for **4** represents a significant barrier to its industrial scaling. However, it is important to note that, although the total synthesis of **4** has not yet been achieved, synthetic routes have been described for structurally related pulvinic acid derivatives [31,32,33], which constitute a valuable basis for future synthetic or semi-synthetic developments.

## 4. Materials and Methods

### 4.1. Lichen Material

Thalli of *Pseudocyphellaria berberina* (G. Forst.) D. J. Galloway & P. James were collected in the Conguillio area IX Region, Chile (38°27′42′′ S, 71°37′48′′ W). Representative specimens are deposited in the Lichen Herbarium of the Escuela de Química y Farmacia, Universidad de Valparaiso.

Taxonomic identification of the species was carried out following dichotomous keys. Characters were observed with a stereoscopic magnifying glass (Zeis Stemi 305, Oberkochen, Germany). In addition, the samples were compared with those from the lichen herbarium. Once collected, the samples were cleaned and dried.

^1^H-NMR and ^13^C-NMR spectra were recorded in CDCl_3_ and are referenced to the residual peaks of CHCl_3_ at δ = 7.26 ppm and δ = 77.0 ppm for ^1^H and ^13^C, respectively, on Avance 400 digital NMR spectrometer (Bruke, Rheinstetten, Germany) operating at 400.1 MHz for ^1^H and 100.6 MHz for ^13^C.

### 4.2. Isolation of Compounds

Brialmontin 2 (**1**), physciosporin (**2**), pseudocyphellarin A (**3**), calycin (**4**), and 22- hydroxistictan-3-one (**5**) were isolated from the lichen material. *P. berberina* (200 g) was extracted in solvents of increasing polarity (hexane-ethyl acetate-acetone). Extraction was performed, assisted by an ultrasound at 40 °C, and 4 pulses of 30 min each were performed with 15 min of cooling between each one. The extract was concentrated under reduced pressure. The dry extract was separated by column chromatography, using silica gel 60 (0.063–0.200 mm) and a mobile phase of increasing polarity (hexane-ethyl acetate) as a stationary phase, and five pure products were obtained. The compounds were obtained in 0.27, 0.34, 1.62, 0.29, and 1.15% yields, respectively. (see spectroscopy in Supplementary Materials).

**Brialmontin 2 (C_18_H_10_O_5_):** white solid (mp 106.3–106.7 °C).

^1^H-RMN (CDCl_3_, 400 MHz) δ: 11.42 (1H, s, OH); 6.65 (1H, s, H-1′); 3.83 (3H, s, OMe-4); 3.73 (3H, s, OMe-2′); 2.54 (3H, s, Me-9′); 2.31 (3H, s, Me-8); 2.23 (3H, s, Me-8′); 2.20 (3H, s, Me-9); 2.03 (6H, s, Me-10, Me-10′).

^13^C-RMN (CDCl_3_, 100 MHz) δ: 170.6 (C7); 162.2 (C2); 161.1 (C4); 155.9 (C2′); 148.2 (C4′); 138.2 (C6); 135.3 (C6′); 122.2 (C5′); 120.3 (C1); 116.7 (C5); 116.2 (C3′); 110.3 (C3); 108.1 (C1′); 60.1 (C4-OMe); 55.7 (C2′-OMe); 29.7 (C7′); 20.4 (C10′); 19.3 (C10); 12.7 (C9)*; 12.5 (C9′)*; 9.6 (C8); 9.1 (C8′).

**Physciosporin (C_19_H_15_ClO_8_):** pale yellow solid (mp 197.6–198.0 °C).

^1^H-RMN (CDCl_3_, 400 MHz) δ: 2.28 (3H, s, Me); 2.58 (3H, s, Me); 2.60 (3H, s, Me); 3.97 (3H, s, COOMe); 10.16 (1H, s, CHO); 11.41 (1H, s, OH); 12.81 (1H, s, OH).

^13^C-RMN (CDCl_3_, 100 MHz) δ: 192.8 (C8-CHO); 171.3 (C7′); 162.7 (C7); 161.3 (C4); 161.0 (C2); 159.3 (C2′); 150.6 (C1); 146.8 (C4′); 142.4 (C5′); 129.2 (C6′); 121.3 (C5); 117.2 (C3′); 114.3 (C6); 110.8 (C3); 109.3 (C1′); 52.6 (COOMe); 19.8 (Me)*; 15.6 (Me)*; 9.3 (C3′-Me). * interchangeable signals.

**Calycin (C_18_H_10_O_5_):** orange needles (mp 249.0–249.5 °C).

^1^H-RMN (CDCl_3_, 400 MHz) δ: 12.59 (1H, s, OH); 8.19 (2H, d, J = 7.8 Hz, H-2″, H-6″); 7.98 (1-H, d, J = 7.3 Hz, H-4); 7.49–7.30 (6H, m, CH ar.); 7.23 (1H, d, 7.3 Hz, H-5).

^13^C-RMN (CDCl_3_, 100 MHz) δ: 173.1 (C7); 165.4 (C12); 160.0 (C10); 153.6 (C1); 131.4 (C5); 129.1 (C4′); 128.6 (C3′/C5′); 128.3 (C1′); 128.2 (C2′)*;128.0 (C6′)*; 125.7 (C3)*; 125.8 (C4)*; 121.7 (C2); 111.1 (C6); 106.3 (C11). * interchangeable signals.

**Pseudocyphellarin A (C_21_H_22_O_8_):** pale yellow solid (mp 173.3–173.9 °C).

^1^H-RMN (CDCl_3_, 400 MHz)δ: 13.08 (1H, s, OH); 12.40 (1H, s, OH); 11.12 (1H, s, OH); 10.38 (1H, s, CHO); 3.98 (3H, s, COOMe); 2.48 (3H, s, Me); 2.19 (3H, s, Me); 2.09 (3H, s, Me); 2.05 (6H, s, Me-8′, Me-10′).

^13^C-RMN (CDCl_3_, 100 MHz) δ: 194.0 (C8); 172.1 (C7′); 169.8 (C7); 166.9 (C2); 166.1 (C4); 158.9 (C2′); 151.5 (C6); 150.1 (C4′); 137.6 (C6′); 120.5 (C5′); 118.2 (C5); 116.2 (C1′); 111.9 (C3′); 107.9 (C3); 102.8 (C1); 52.3 (7-COOMe); 20.5 (C10′); 18.9 (C10); 13.2 (C-9′); 10.8 (C9); 9.8 (C8′).

**22-hydroxystictan-3-one (C_30_H_50_O_2_):** white needles (mp 219.5–220.0 °C).

^1^H-RMN (CDCl_3_, 400 MHz) δ: 3.14 (1H, d, J = 11.0 Hz, H-22); 1.16 (3H, s, Me-26); 1.05 (3H, s, Me-23); 1.04 (3H, s, Me-24); 1.01 (3H, s, Me-30); 0.97 (3H, s, Me-27); 0.86 (3H, s, Me-29); 0.74 (3H, s, Me-25); 0.72 (3H, s, Me-28).

^13^C-RMN (CDCl_3_, 100 MHz) δ: 220.4 (C3); 177.3 (C22); 13.5 (C28); 23.3 (C25); 17.1 (C27); 18.5 (C29); 29.7 (C30); 19.5 (C24); 29.3 (C23); 22.0 (C26).

### 4.3. Photoprotective Activity

#### 4.3.1. UVB Photoprotective Activity

The UVB photoprotective activity of lichen metabolites **1** to **5**, and **AE** was determined by the in vitro screening method of Mansur et al., (1986) [34], as described by Nuñez-Arango et al. [35] and Panyakaew et al. [25], estimating the sun protection factor (SPF). Avobenzone (AVO, UVA filter), Eusolex 4360 (Eu4360, UVA-UVB filter), and 2-phenyl-5-bencimidazolesulphonic acid (PBSA, UVB filter) (Sigma-Aldrich, St. Louis, MO, USA) were the controls. EtOH solutions of the samples (10, 50, 100, and 200 ppm) were spectrophotometrically scanned (290–400 nm, intervals of 1 nm) in a UV-VIS GENESYS 10 (Thermo Scientific, Madison, WI, USA) using quartz cells (1 cm) and absolute EtOH as a blank. SPF was calculated as indicated by Equation (1):

(1) $$SPF=CF\times \sum_{290}^{320}EE\left(\lambda \right)\times I\left(\lambda \right)\times Abs\left(\lambda \right)$$

where EE (λ) = the erythemal effect spectrum; I (λ) = the solar intensity spectrum; Abs (λ) = the absorbance of the evaluated substance; CF= the correction factor (=10); and EE × I = are constant values [36].

Different photoprotection levels are set based on SPF values: 2 to 15 (low); 15 to 30 (medium); 30 to 50 (high); and >50 (highest) [37].

#### 4.3.2. UVA Photoprotective Activity

The UVA photoprotective activity of **1** to **5**, and **AE** was determined by calculating the critical wavelength (λ_crit_) and the UVA/UVB ratio (UVA/UVB-r). λ_crit_ was determined with the absorbance data of the compound solutions (at 100 ppm) using Equation (2):

(2) $$\int_{290\;nm}^{\lambda crit}Abs\left(\lambda \right)\;d\lambda =0.9\times \int_{290\;nm}^{400\;nm}Abs\left(\lambda \right)\;d\lambda$$

where Abs = the absorbance of the compound solution and λ_crit_ = the wavelength at 90% of the area under the absorbance curve (AUC) from 290 to 400 nm. The AUC was 100%.

According to the λ_crit_, different levels of a UVA broad spectrum rating can be set: λ_crit_ < 325 nm (0 star); 325 to 335 nm (*); 335 to 350 nm (**); 350 to 370 nm (***); and ≥370 nm (****) [25,26].

In turn, the UVA/UVB-r, considered as the ratio of mean UVA absorbance to mean UVB absorbance, was determined using the absorbance data (at 100 ppm) according to Equation (3):

(3) $$UVA/UVB\;ratio=\frac{\int_{320\;nm}^{400\;nm}Abs\left(\lambda \right)d(\lambda )/\int_{320\;nm}^{400\;nm}d(\lambda )}{\int_{290\;nm}^{320\;nm}Abs\left(\lambda \right)d(\lambda )/\int_{290\;nm}^{320\;nm}d(\lambda )}$$

According to the UVA/UVB-r values and UVA star rating, different levels of photoprotection can be set: UVA/UVB-r < 0.2 (0 star and no UVA protection); 0.2 to 0.4 (* or “moderate”); 0.4 to 0.6 (** or “good”); 0.6 to 0.8 (*** or “superior”); and ≥0.8 (**** or “maximum”) [26,35].

### 4.4. Antioxidant Activity

Compounds **1** to **5** and **AE** were tested in vitro using DPPH (2,2-diphenyl-1-picrylhydrazyl) free radical assay according to the protocol described previously [38]. The stock solution of ascorbic acid (4.1 mg), trolox (4.3 mg), pseudocyphellarin A (4.3 mg), 22-hydroxyestictan-3-one (4.3 mg), brialmontin 2 (4.3 mg), physciosporin (4.1 mg), acetone extract (4.3 mg) was prepared in 25 mL of ethanol. A total of 1 mL of the sample was mixed with 3 mL of the DPPH solution (0.25 mg/L). After 30 min of reaction (at room temperature in the dark), the absorbance was measured at 517 nm in the spectrophotometer UV-VIS GENESYS 10 (Thermo Scientific, Madison, WI, USA). Free radical scavenging activity (*RSA*) (%) was calculated using Equation (4):

(4) $$RSA\;\left(\%\right)=100\;\frac{\left(A_{blank}-A_{sample}\right)}{\left(A_{blank}\right)}$$

where RSA = free radical scavenging activity; A_blank_ = the absorbance of the blank (solvent mixture instead of sample solution); and A_sample_ = the absorbance of the sample.

### 4.5. Cytotoxicity Assay

#### 4.5.1. Cell Culture

Human epidermal keratinocytes (HEKs; Thermo Fisher Scientific, San Diego, CA, USA) were cultured in an EpiLife™ medium (Thermo Fisher Scientific, San Diego, CA, USA) supplemented with a Human Keratinocyte Growth Supplement (HKGS; Thermo Fisher Scientific, San Diego, CA, USA) and 1% penicillin-streptomycin (Gibco, Thermo Fisher Scientific, San Diego, CA, USA). Cells were maintained at 37 °C in a humidified atmosphere containing 5% CO_2_. Subcultures were performed at approximately 80% confluence, and cells between passages 2 and 4 were used for experiments.

#### 4.5.2. Treatment with Lichen Compounds and Extract

HEK cells were seeded in flat-bottom 96-well plates (Corning, NY, USA) at a density of 15,000 cells/well in 100 μL of a complete EpiLife™ medium and incubated for 24 h to allow cell adhesion. Cells were then treated with 10, 50, 100, or 200 ppm of each compound or extract, prepared in culture medium. Negative controls (untreated cells) and positive controls (cells treated with 0.1% Triton X-100; Sigma-Aldrich, St. Louis, MO, USA) were included. All conditions were tested in triplicate wells (technical replicates), and experiments were repeated three times independently (biological replicates).

#### 4.5.3. Resazurin Reduction Assay

After 24 h of treatment, 10 μL of a resazurin sodium salt solution (0.01% *w*/*v* in sterile phosphate-buffered saline, PBS; Sigma-Aldrich, St. Louis, MO, USA) was added to each well. The plates were incubated for 2 h at 37 °C in the dark. Fluorescence was measured using a Varioskan LUX multimode microplate reader (Thermo Fisher Scientific, San Diego, CA, USA) at 560 nm excitation and 590 nm emission wavelengths. Fluorescence intensity was proportional to the number of metabolically active, viable cells.

### 4.6. In Silico Estimation of Skin Permeation and Application of Metabolites 1 to 4 as Topical Dermatological Agents

The physicochemical parameters indicative of skin permeation and the possible application of metabolites **1** to **4** as topical dermatological agents [27,29] were calculated in silico using the SwissADME© online platform [39]. Gibbs free energy of transfer from water to *n*-octanol (Δ_t_G°) at 298.15 K was calculated according to Equation (5):

∆_t_G° = −RT Ln P (5)

where R = the ideal molar gas constant (8.314 J mol^−1^ K^−1^), and T = the absolute temperature in kelvin.

### 4.7. Statistical Analysis

Statistical analysis was performed using one-way variance analyses (ANOVA) and Tukey’s post-hoc or Tukey’s multiple comparison tests using Real Statistics Resource Pack software (release 8.9.1), Copyright (2013–2023), and GraphPad Prism version 10.0 (GraphPad Software, San Diego, CA, USA). Differences were considered statistically significant at *p* < 0.05. Data are shown as the mean ± the standard deviation (sd) [40].

## 5. Conclusions

Brialmontin 2 (**1**), physciosporin (**2**), and pseudocyphellarin A (**3**) were isolated for the first time from the lichen *Pseudocyphellaria berberina* (G. Forst.) D. J. Galloway & P. James, along with calycin (**4**) and 22-hydroxystictan-3-one (**5**). The acetone extract (**AE**) and metabolites **2** and **4** are photoprotectors with UVB and UVA properties, whereas **1** and **3** are UVA photoprotectors. Additionally, **AE** and **4** showed antioxidant activity; therefore, they are dual agents with both properties. In addition, **4** maintains 70% viability in HEK cells at concentrations of 100 ppm. The in silico parameters suggest that 4 spontaneously diffuses into the stratum corneum with limited absorption through the skin. Therefore, it could be considered as an active ingredient for sunscreen or dermo-cosmetic formulations.

## Figures and Tables

**Table 1:** UVA photoprotective activity of extract of *P. berberina* and its isolated metabolites **1** to **5**.

	UVA Photoprotection ^a^	
	λ_crit._ (nm) ± SD	UVA/UVB-r ± SD
PBSA	326.80 (0.44) *	0.141 (0.004) no UVA
AVO	378.44 (0.22) ****	1.740 (0.173) **** maximum
Eu4360	353.19 (1.68) ***	0.452 (0.009) ** good
AE	389.09 (0.84) ****	1.118 (0.010) **** maximum
1	342.51 (1.15) **	0.308 (0.002) * moderate
2	358.33 (0.32) ***	0.425 (0.006) ** good
3	364.68 (2.29) ***	0.549 (0.003) ** good
4	391.08 (0.35) ****	1.353 (0.031) **** maximum
5	NA ^b^	NA ^b^

^a^ λ_crit_ and UVA/UVB-r were determined at 100 ppm. Star rating for λ_crit_: <325 nm (zero star); 325 to 335 nm (*); 335 to 350 nm (**); 350 to 370 nm (***); ≥370 nm (****). A higher number of stars higher UVA photoprotective capacity [25,26]. Boots stars system according to UVA/UVB ratio: UVA/UVB-r < 0.2 (zero star and no UVA protection); 0.2 to 0.4 (* or “moderate”); 0.4 to 0.6 (** or “good”); 0.6 to 0.8 (*** or “superior”); and ≥0.8 (**** or “maximum”) UVA photoprotective capacity [25,26]. Each value is the mean of three independent experiments ± standard deviation (sd). ^b^ Non active.

**Table 2:** In silico estimation of possible skin permeation and application of **1** to **4** as topical dermatological agents and photoprotective controls.

Sample	Skin Permeability Coefficient	Partition Coefficient (P)	Gibbs Free Energy of Transfer	Polar Surface Area	Molecular Weight	# Aromatic Rings
	Log *k_p_* (cm s^−1^)	cLog P	Δ_t_G° (kJ mol^−1^)	TPSA Å^2^	MW Da	Ar#
**PBSA**	–6.56	2.34	–13.37	91.43	274.30	3
**Eu4360**	–5.00	2.75	–15.71	46.53	228.24	2
**AVO**	–4.81	4.07	–23.23	43.37	310.39	2
**1**	–4.47	4.57	–26.11	64.99	358.43	2
**2**	–5.68	3.33	–19.02	119.36	406.77	3
**3**	–5.16	3.40	–20.60	130.36	402.39	2
**4**	–6.31	2.61	–14.87	72.83	306.27	4

Photoprotectors Eu4360 and AVO comply with four physicochemical parameters (cLog P 1 to 4, TPSA ≤ 100 Å^2^, MW < 500 Da and Ar# ≤ 2), whereas PBSA complies with three (cLog P, TPSA and MW). Compounds **1** (TPSA, MW and Ar#), **3** (cLog P, MW and Ar #), and **4** (cLog P, TPSA and MW) comply with three parameters, whereas **2** only complies with two (cLog P and MW). Therefore, **1**, **3,** and **4** could be added as photoprotective ingredients for sunscreens formulations.

## Data Availability

The data presented in this study are available upon request from the corresponding author.

## Supplementary Materials

The following supporting information can be downloaded at https://www.mdpi.com/article/10.3390/molecules30183833/s1: NMR spectroscopy. Figure S1: The ^1^H NMR spectrum of compound 1, Figure S2: The ^13^C NMR spectrum of compound **1**, Figure S3: The ^1^H NMR spectrum of compound 2, Figure S4: The ^13^C NMR spectrum of compound **2**, Figure S5: The ^1^H NMR spectrum of compound 3, Figure S6: The ^13^C NMR spectrum of compound **3**, Figure S7: The ^1^H NMR spectrum of compound 4, Figure S8: The ^13^C NMR spectrum of compound **4**, Figure S9: The ^1^H NMR spectrum of compound 5, and Figure S10: The ^13^C NMR spectrum of compound **5**.
