# Supplementary material for: Antioxidant and Photoprotective Capacity of Secondary Metabolites Isolated from Pseudocyphellaria berberina
Source: Molecules. 2025 Sep 22;30(18):3833. doi: 10.3390/molecules30183833 (PMC12472422; doi:10.3390/molecules30183833)
Supplement: Supplementary file 1 [file molecules-30-03833-s001.zip › molecules-3806057-supplementary.pdf]

Antioxidant and Photoprotective Capacity of Secondary Metabolites Isolated from *Pseudocyphellaria berberina*

Cecilia Rubio [1,2], Javiera Ramírez [1], José L. Rojas [3], Norma A. Valencia-Islas [4], Carolina Campos[5,6] and Natalia Quiñones [1,6*]

[1] Herbario de Líquenes, Escuela de Química y Farmacia, Facultad de Farmacia, Universidad de Valparaíso, Valparaíso, 2340000, Chile cecilia.rubiol@uv.cl (C.R.); natalia.quinones@uv.cl (N.Q.); javiera.ramirez@uv.cl (J.R.P.)

[2] Magíster en Gestión Farmacéutica y Farmacia Asistencial, Escuela de Química y Farmacia, Facultad de Farmacia, Universidad de Valparaíso. Cecilia.rubiol@uv.cl

[3] Departamento de Química, Facultad de Ciencias, Universidad Nacional de Colombia, Sede Bogotá, 111321, Colombia; jlrojasa@unal.edu.co (J.R.)

[4] Departamento de Farmacia, Facultad de Ciencias, Universidad Nacional de Colombia, Sede Bogotá, 111321, Colombia; navalenciai@unal.edu.co (N.V.)

[5] Laboratorio de toxicología (LADETOX), Escuela de Química y Farmacia, Facultad de Farmacia, Universidad de Valparaíso, Valparaíso, 2340000, Chile. carolina.campos@uv.cl (C.C.)

[6] Centro de Investigación, Desarrollo e Innovación de Productos Bioactivos (CInBIO), Universidad de Valparaíso, Valparaíso, 2340000, Chile. natalia.quinones@uv.cl (N.Q.); carolina.campos@uv.cl (C.C.)

* Correspondence: natalia.quinones@uv.cl

Supporting Information

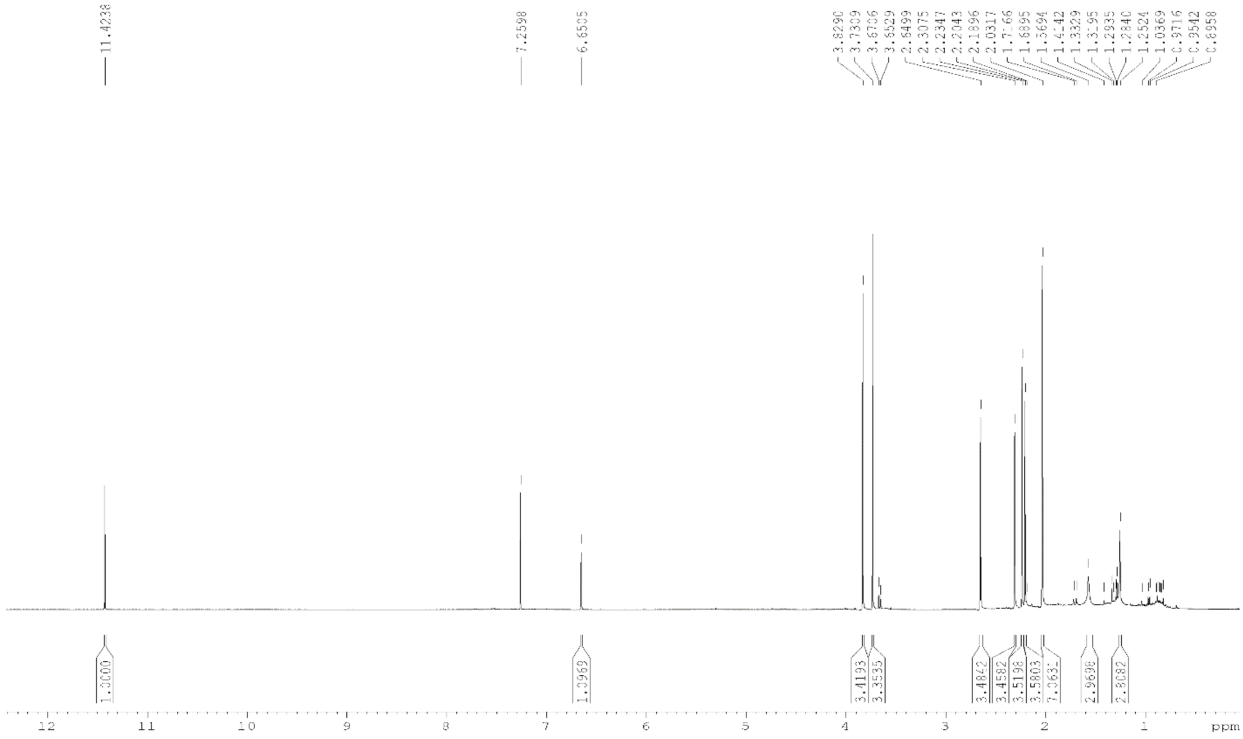

Figure S1: The ^{1}H NMR spectrum of compound **1.**

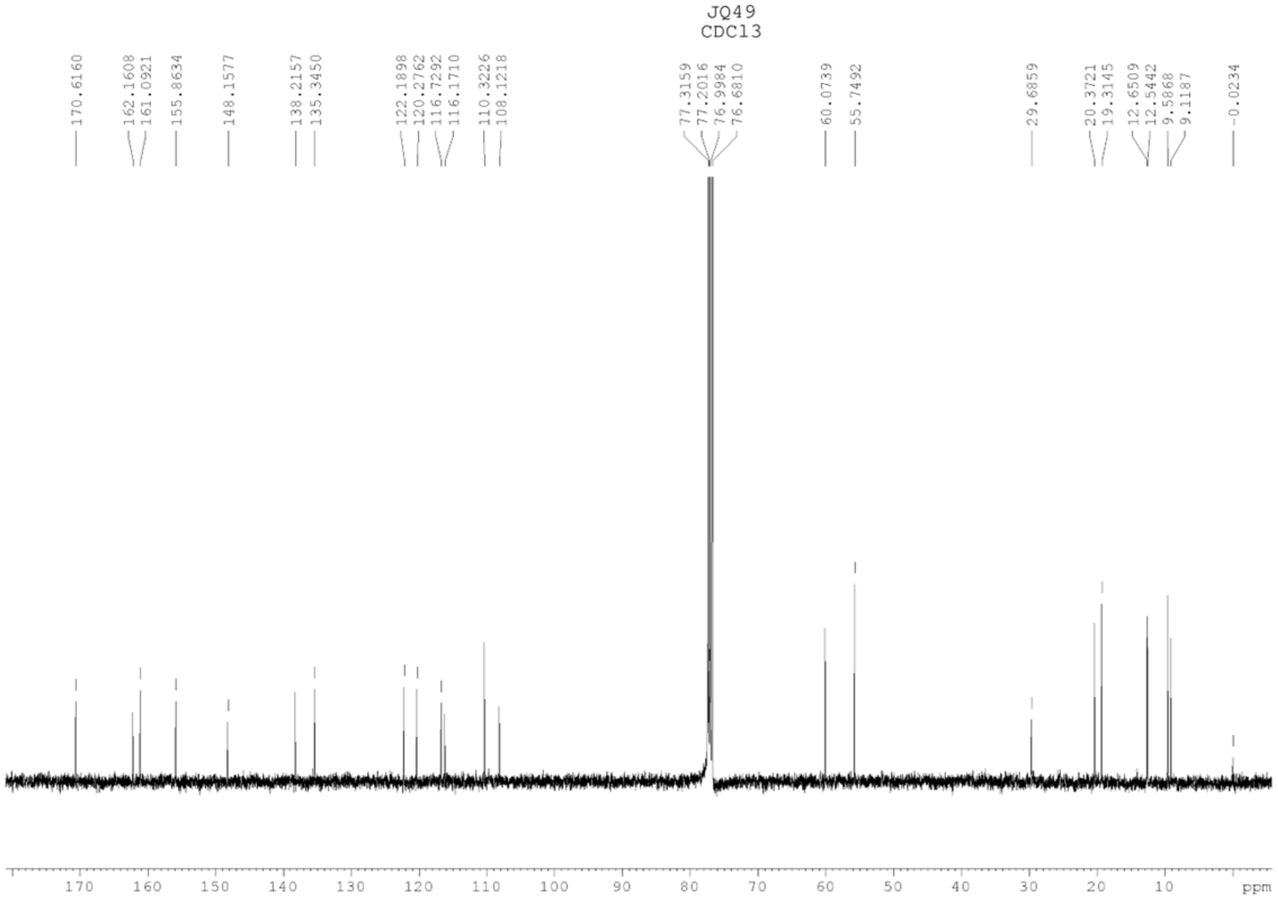

Figure S2: The ^{13}C NMR spectrum of compounds **1.**

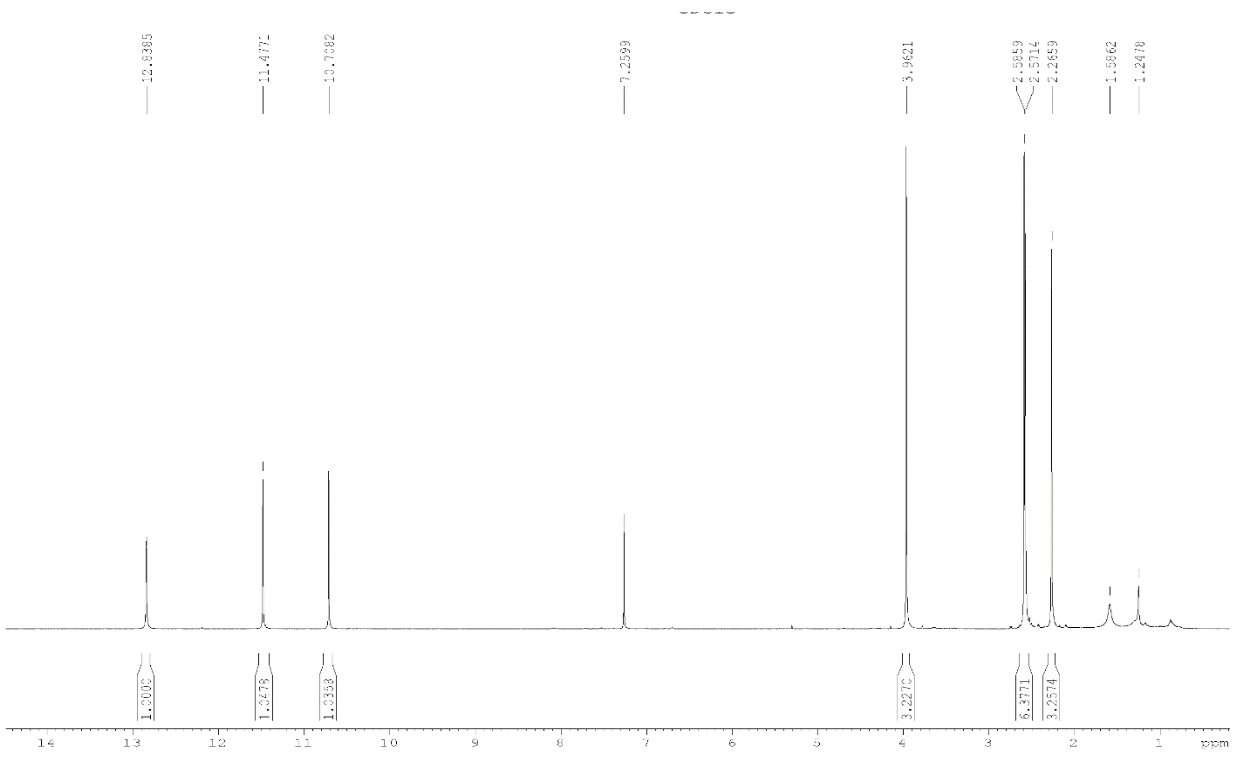

Figure S3: The ^{1}H NMR spectrum of compound **2.**

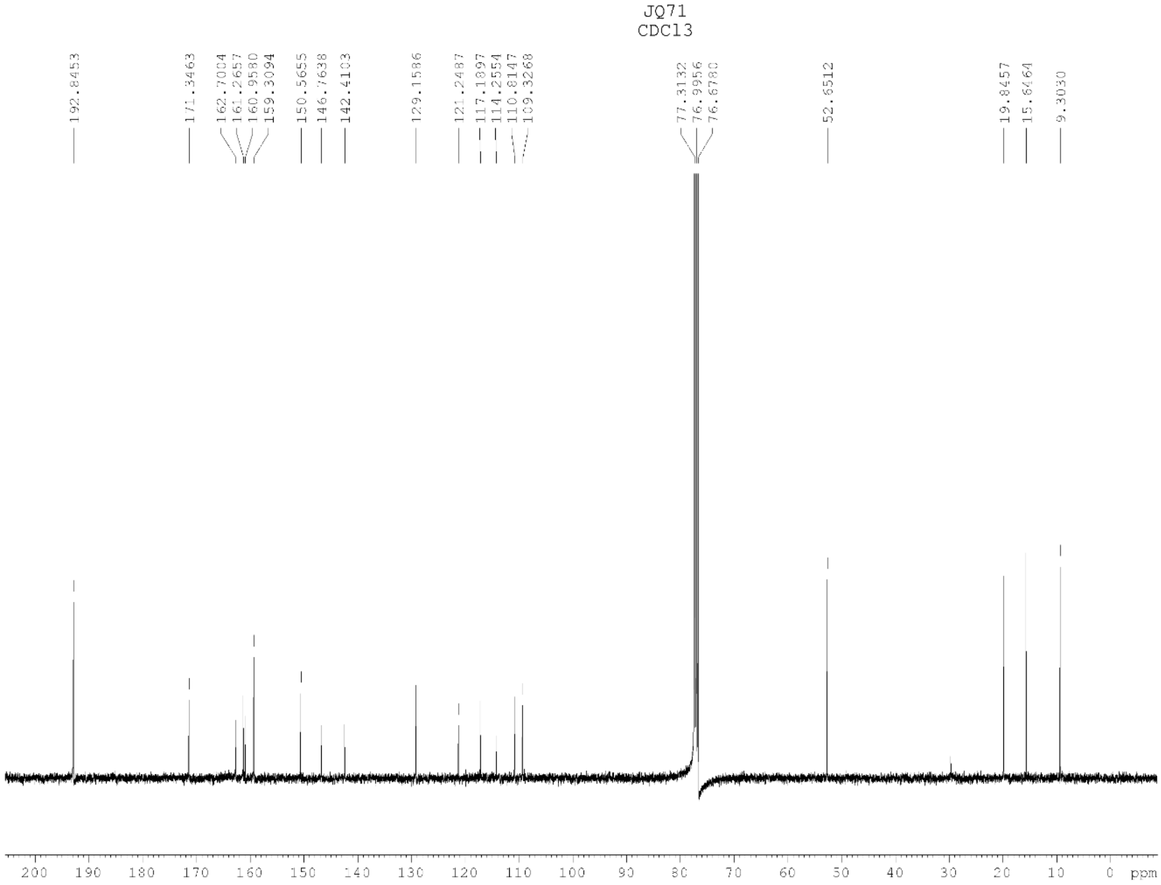

Figure S4: The ^{13}C NMR spectrum of compound **2**.

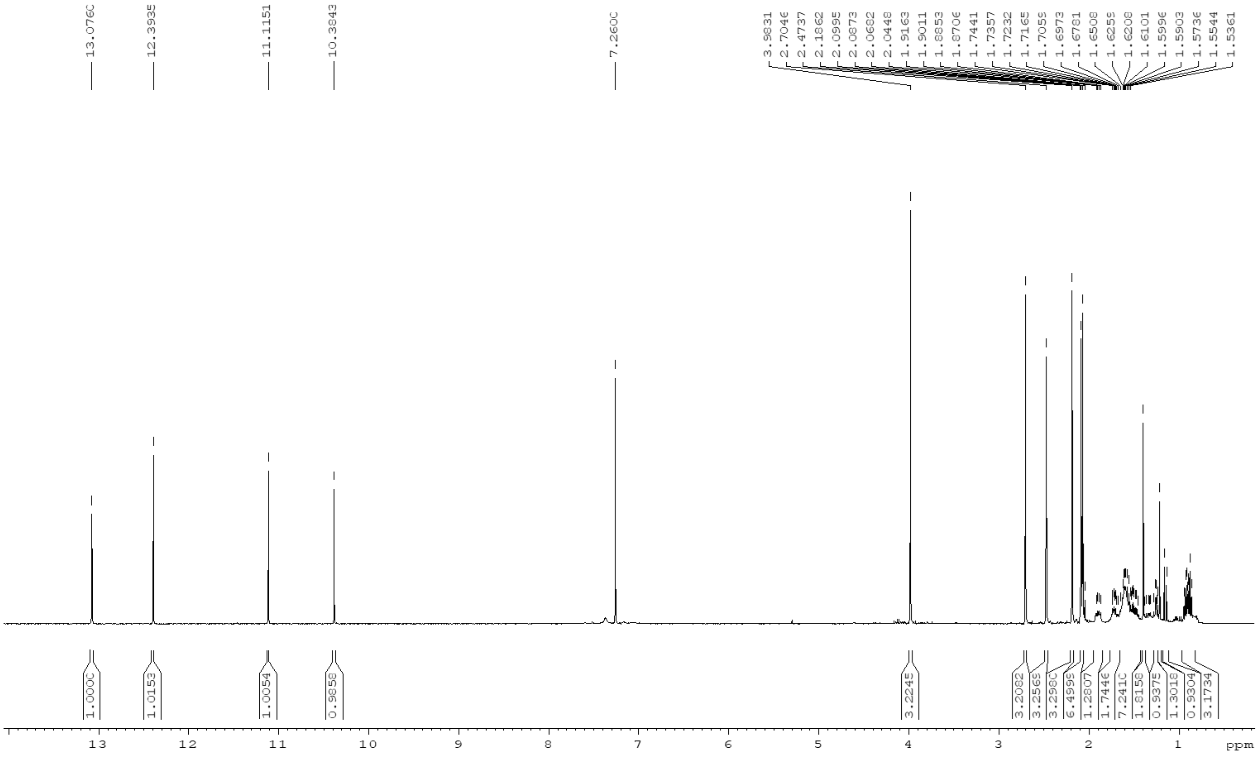

Figure S5: The ^{1}H NMR spectrum of compound **3.**

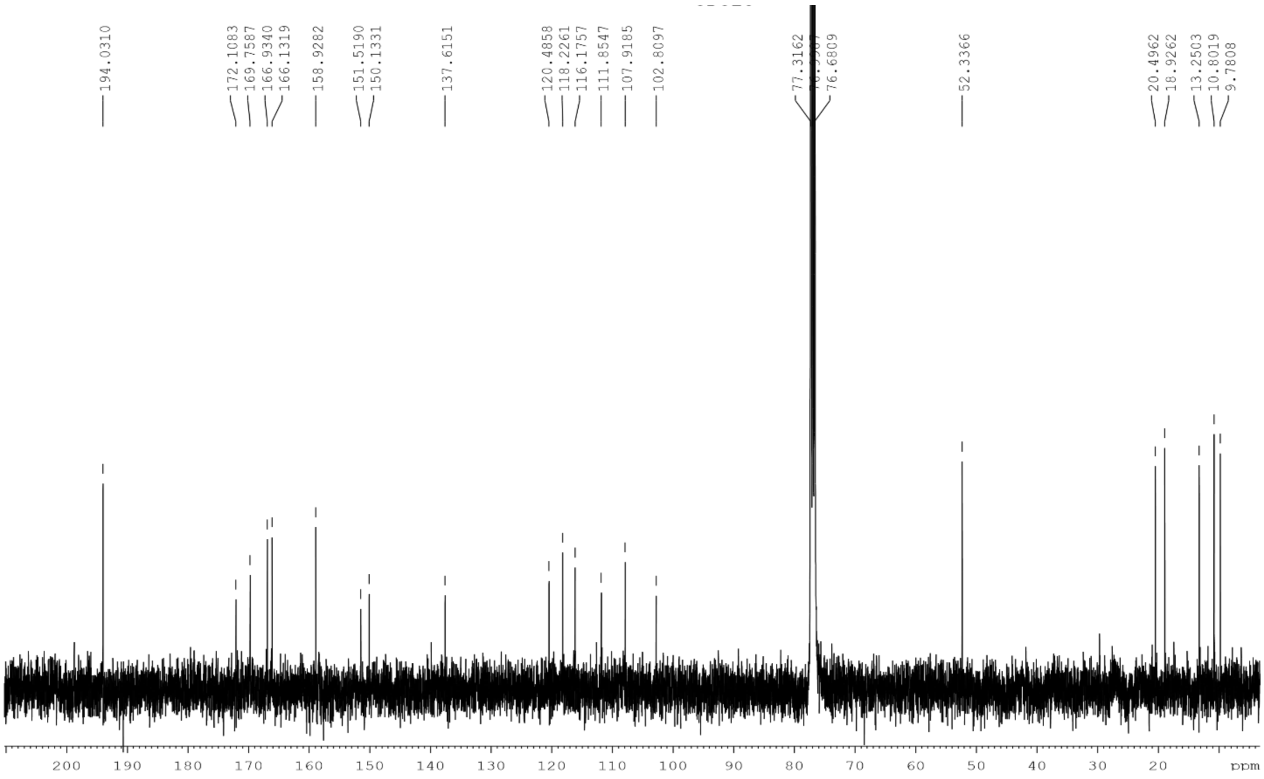

Figure S6: The ^{13}C NMR spectrum of compound **3.**

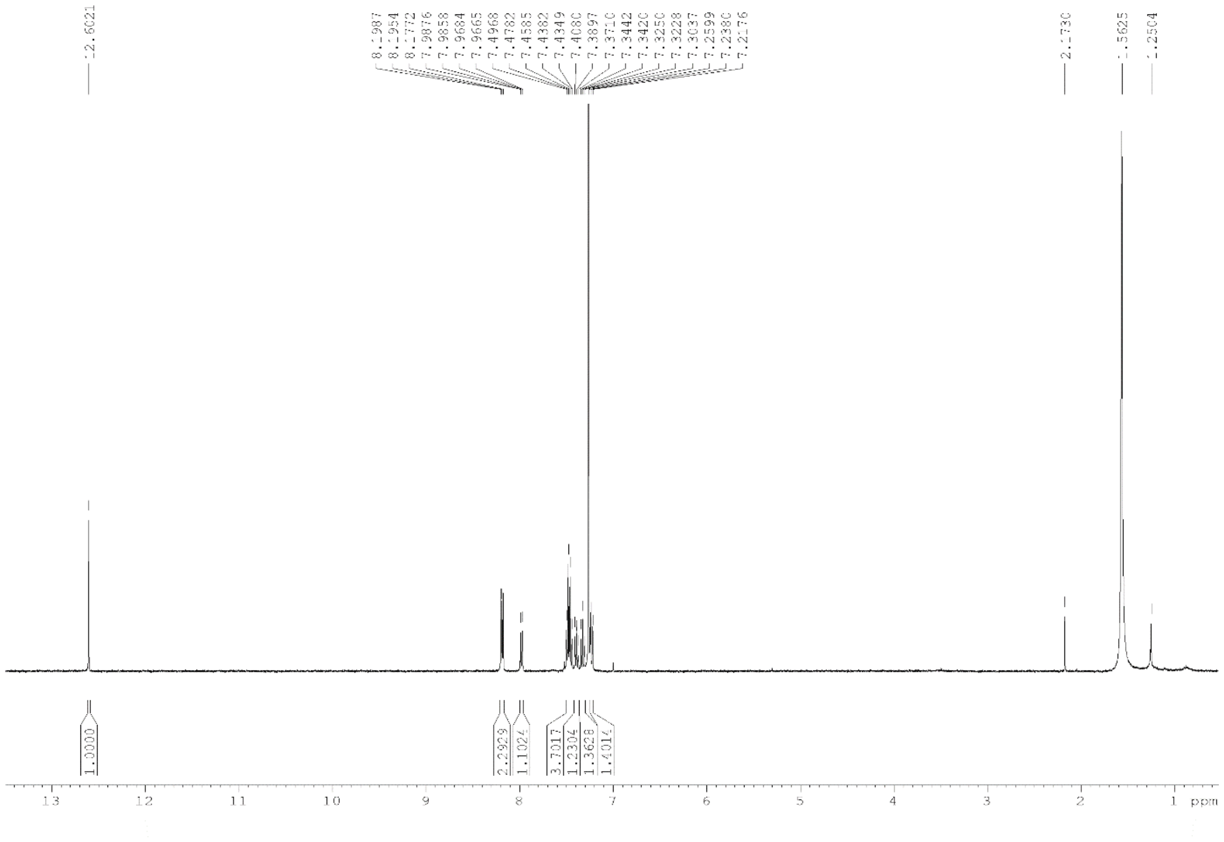

Figure S7: The ^{1}H NMR spectrum of compound **4.**

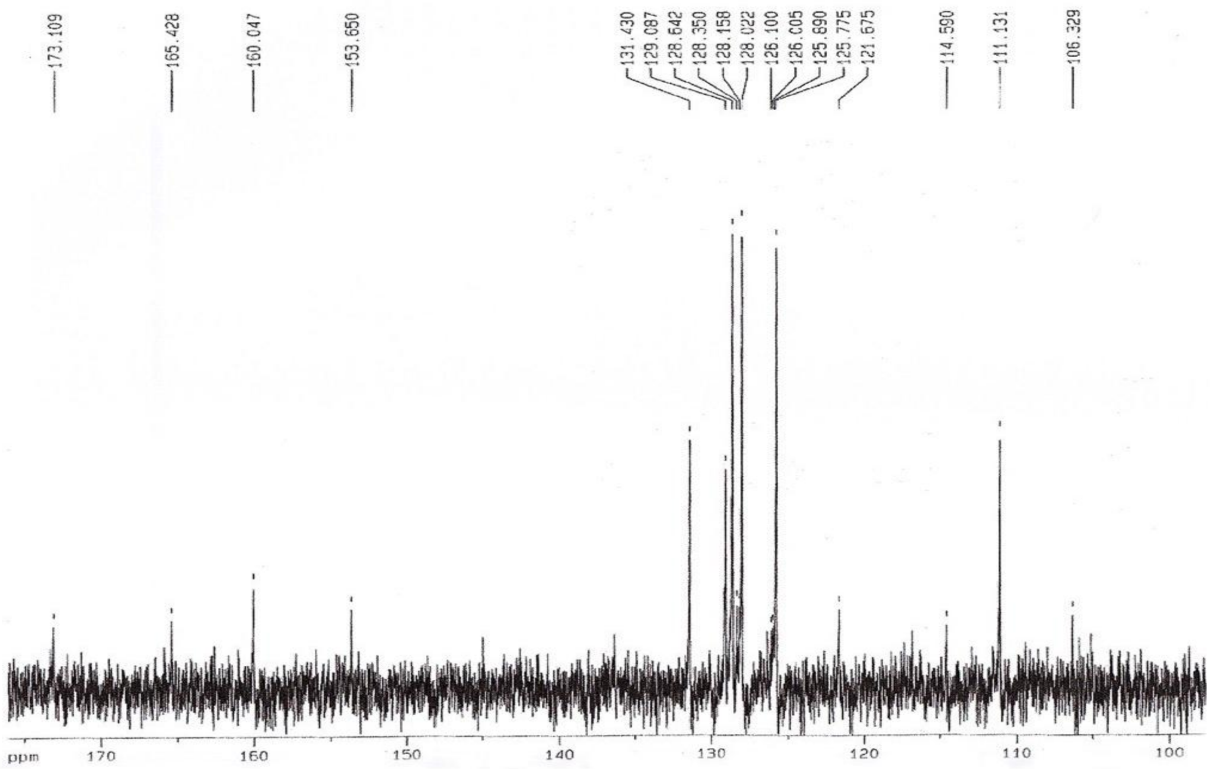

Figure S8: The ^{13}C NMR spectrum of compound **4.**

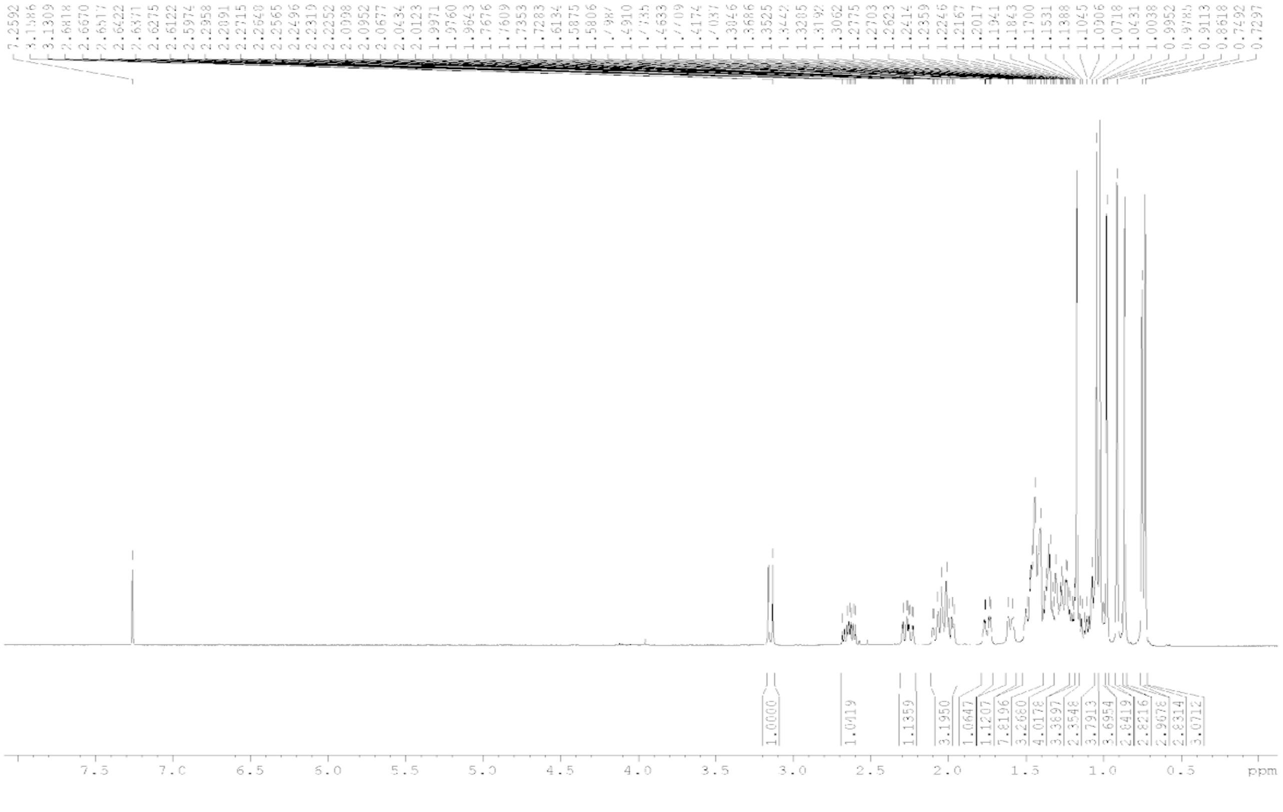

Figure S9: The ^{1}H NMR spectrum of compound **5**

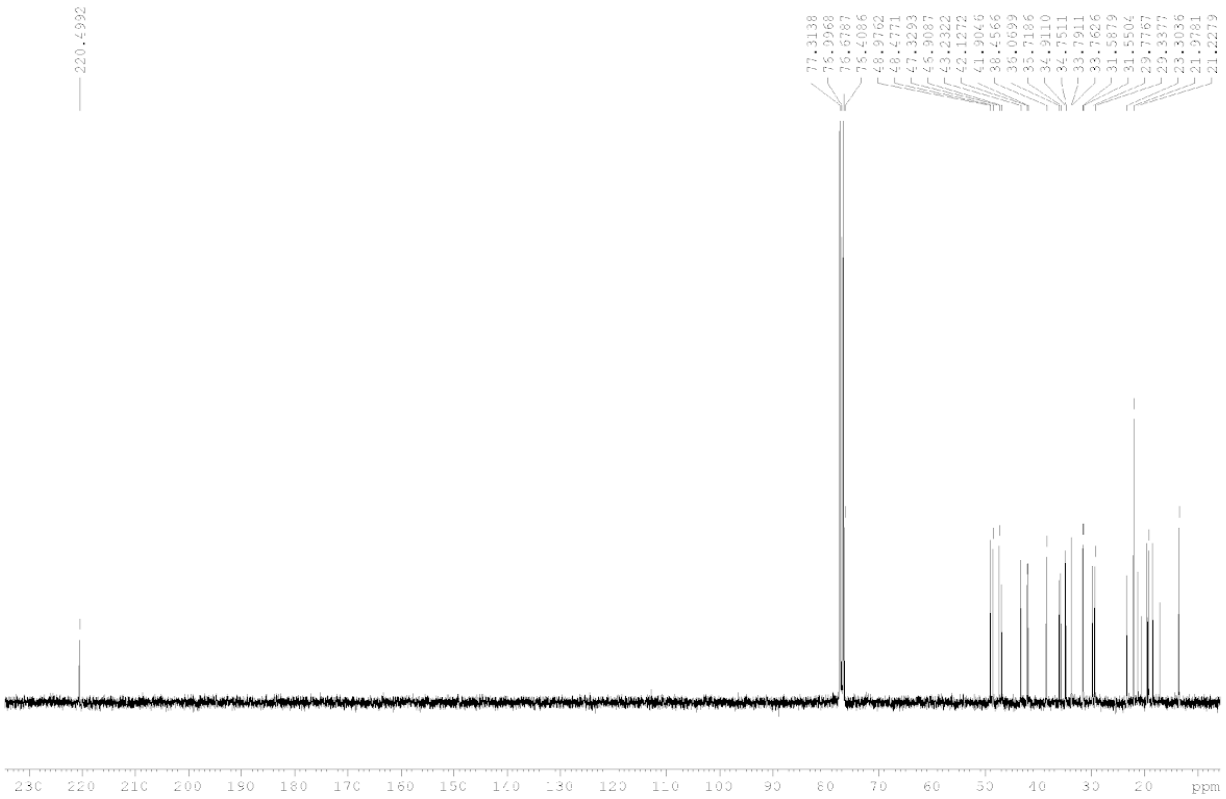

Figure S10: The ^{13}C NMR spectrum of compound **5.**
